# The safety and efficacy of intravenous administration of tranexamic acid in off-pump coronary artery bypass grafting: a systematic review and meta-analysis

**DOI:** 10.3389/fmed.2025.1643712

**Published:** 2025-09-05

**Authors:** Zhi-yao Zou, Jin-rui Song, Qing-Hui Zhang, Xiao-Jin Huang, Yun-tai Yao

**Affiliations:** ^1^Fuwai Yunnan Hospital, Chinese Academy of Medical Sciences, Affiliated Cardiovascular Hospital of Kunming Medical University, Kunming, Yunnan, China; ^2^Department of Anesthesiology, Qujing Maternal and Child Health-care Hospital, Qujing, Yunnan, China; ^3^Department of Anesthesiology, Fuwai Hospital, National Center for Cardiovascular Diseases, Peking Union Medical College and Chinese Academy of Medical Sciences, Beijing, China; ^4^Department of Anesthesiology, Critical Care and Pain Medicine, Center of Outcomes Research, University of Texas, Houston, TX, United States; ^5^Outcomes Research Consortium, Houston, TX, United States

**Keywords:** coronary artery bypass, off-pump, tranexamic acid, postoperative complications, coagulation

## Abstract

**Study objective:**

To assess the efficacy and safety of tranexamic acid (TXA) on off-pump coronary artery bypass (OPCAB) surgery.

**Design:**

Meta-analysis.

**Methods:**

Relevant trials were identified by computerized searches of PUBMED, Cochrane Library, EMBASE, OVID, China National Knowledge Infrastructure (CNKI), Wanfang Data and VIP Data till Aug 8th, 2025, were searched using search terms “Tranexamic acid,” “coronary artery bypass grafting,” “off-pump,” “randomized controlled trial” database search was updated on Aug 10th, 2025. Primary outcomes included intraoperative and postoperative bleeding.

**Results:**

Nineteen randomized controlled trials were finally included in the current study. Intravenous TXA reduced intraoperative and postoperative bleeding volume (including 2, 4, 6, and 24-h postoperative bleeding). It also decreased the rate and volume of red blood cell (RBC) and fresh frozen plasma (FFP) transfusions, with no effect on reoperation rates due to postoperative bleeding. At 24 h postoperatively, TXA increased platelet counts, hemoglobin concentrations, and prothrombin time (PT), while decreasing activated partial thromboplastin time (APTT), fibrinogen levels, and D-dimer concentrations. Importantly, TXA did not elevate the risk of postoperative complications (e.g., mortality, myocardial infarction, cerebrovascular accidents, thrombotic events) and had no impact on levels of CK-MB, creatinine, interleukin-6, or lengths of intensive care unit (ICU) and hospital stays.

**Conclusion:**

Intravenous TXA is effective in reducing perioperative bleeding and transfusion requirements in OPCAB without increasing the risk of major postoperative complications, supporting its clinical utility in this setting. More well-designed and adequately powered RCTs are needed to confirm this further.

## Highlights

- Of the 19 included randomized controlled trials (RCTs), 8 were identified from Chinese databases and 11 from international sources, with searches conducted across both Chinese and English databases.
- The outcome of our current study was relatively comprehensive.
  **Primary outcomes** were defined as: Intraoperative bleeding volume (measured. in mL). Postoperative bleeding volume at 2, 4, 6, and 24 h (measured in mL). Red blood cell (RBC) transfusion rate (percentage of patients requiring RBC transfusion postoperatively).
  **Secondary outcomes** included transfusion volumes of RBCs, fresh frozen plasma (FFP), and platelet concentrates (PC). Postoperative 24-h laboratory parameters: platelet count, hemoglobin concentration, prothrombin time (PT), activated partial thromboplastin time (APTT), fibrinogen level, and D-dimer concentration.
- **Question**
  The efficacy and safety of intravenous administration of TXA in OPCAB remains unconfirmed.
- **Findings**
- Intravenous TXA reduced intraoperative and postoperative bleeding volume (including 2, 4, 6, and 24-h postoperative bleeding). It also decreased the rate and volume of RBC and FFP transfusions, with no effect on reoperation rates due to postoperative bleeding.
- Intravenous TXA had no different effects on reoperation due to postoperative bleeding.
- At 24 h postoperatively, intravenous TXA increased platelet counts, hemoglobin concentrations, and prothrombin time (PT), while decreasing activated partial thromboplastin time (APTT), fibrinogen levels, and D-dimer concentrations.
- Importantly, intravenous TXA did not elevate the risk of postoperative complications (e.g., mortality, myocardial infarction, cerebrovascular accidents, thrombotic events).
- Intravenous TXA had no impact on levels of CK-MB, creatinine, interleukin-6, or lengths of intensive care unit (ICU) and hospital stays.
- **Meaning**
- Intravenous TXA reduced both intraoperative and postoperative bleeding volume, as well as RBC/FFP transfusion rate and volume at postoperative. More well-designed and adequately powered RCTs are needed to confirm this further.

## 1 Introduction

Perioperative bleeding and coagulopathy are major complications of coronary artery bypass graft (CABG) surgery. To reduce the morbidity associated with cardiopulmonary bypass, off-pump CABG (OPCAB) surgery has gained popularity. However, even without cardiopulmonary bypass, the fibrinolytic pathway is activated because of the surgical trauma and exposure to heparin and protamine. Additionally, greater activation of fibrinogen associated with OPCAB surgery than on-pump CABG surgery might result in a higher incidence of adverse thrombotic events (1, 2).

Tranexamic acid (TXA) is an analog of lysine and acts primarily to block the lysine binding site on plasminogen molecules, preventing plasmin formation and thus inhibiting fibrinolysis. TXA has been applied in various types of surgery for hemostasis purposes, including orthopedic surgeries (total hip arthroplasty), cardiac surgery, cerebral surgery, etc. (3, 4). Dai et al. (5) meta-analysis aimed to clarify the efficacy and safety of using TXA to reduce blood loss during OPCAB surgery. However, the authors included studies examining intrapleural and intravenous TXA use in reducing blood transfusion. As a result, the efficacy of intravenous TXA in OPCAB surgery scenarios remains inconclusive. Sun et al. (6) conducted a meta-analysis to evaluate the efficacy and safety of TXA in OPCAB surgery, focusing solely on intravenous administration; however, the endpoints include only blood product transfusion rate, postoperative death, and thrombotic events. Moreover, previous meta-analyses that have been published did not incorporate trials from Chinese databases.

Previous studies from our team have shown that TXA can reduce perioperative bleeding and blood product transfusion in patients undergoing cardiac surgery and have different effects on perioperative myocardial enzymes (7), inflammatory factors (8), and platelet counts and functions in patients undergoing cardiac surgery. TXA had an anti-inflammatory effect in adult cardiac surgery patients (8), and TXA administration was associated with less myocardial injury in cardiac surgery patients (7). A multicenter, double-blind, randomized clinical trial among adult patients undergoing cardiac surgery with cardiopulmonary bypass (9). The study enrolled 3,079 patients at four hospitals in China. Participants received either a high-dose or low-dose tranexamic acid. TXA infusion resulted in a modest statistically reduction in the proportion of patients who received allogeneic red blood cells transfusion and met criteria for noninferiority with respect to a composite primary safety endpoint consisting of 30-day mortality, seizure, kidney dysfunction, and thrombotic events. However, the safety of TXA used in OPCAB surgery remains controversial, as there are limited randomized controlled trials (RCTs) studying the effects on the efficacy of transfusion rates and volumes, postoperative blood loss, and the risk of thrombotic events, myocardial infarction, and cerebrovascular accident.

In this study, we included RCTs from both Chinese and English databases (PubMed, Cochrane Library, CNKI, etc.). A total of 19 trials were included in this study, of which 8 were from Chinese databases. This study aimed to provide a meta-analysis including 19 randomized controlled trials (10–28) to more comprehensively evaluate the efficacy and safety of TXA in OPGABG, focusing solely on intravenous administration.

## 2 Methods and methods

### 2.1 Ethical approval

This study analyzed previously published literatures; ethical approval was not necessary under the Ethical Committees of Fuwai Hospital and Fuwai Yunnan Cardiovascular Hospital.

### 2.2 Search strategy

We conducted a systemic review according to the Preferred Reporting Items for Systemic Reviews and Meta-Analysis Quality of Reporting of Meta-analysis (PRIMSA) Guidelines (29). The protocol of the current meta-analysis was published in PROSPERO with the registration number CRD42024557846, https://www.crd.york.ac.uk/PROSPERO/view/CRD42024557846. Relevant trials were identified by computerized searches of PUBMED, EMBASE, Cochrane, China National Knowledge Infrastructure (CNKI), Wanfang Data, and VIP Data till Aug 8th, 2025, using different combinations of search words as follows: (tranexamic acid) AND (off pump) AND (coronary artery bypass surgery) AND (randomized controlled trial OR controlled clinical trial OR randomized OR placebo OR randomly OR trial) (Supplementary Table 1). No language restriction was used. Additionally, we used the bibliography of retrieved articles to identify relevant studies further. Database search was updated on Aug 10th, 2025.

### 2.3 Inclusion and exclusion criteria

The authors included all RCTs comparing the effects of intravenous administration of TXA on OPCAB surgery with control. Exclusion criteria include (1) studies published as review articles, case reports, or abstracts; (2) studies based on animal models; (3) duplicate publications; (4) studies lacking information about outcomes of interest; (5) control groups were restricted to placebo (saline) or standard care without antifibrinolytics. Two authors (Z. Y. Z. and Y. T. Y.) independently review the titles and abstracts of all identified studies for eligibility, excluding ineligible ones. The eligibility of those remaining studies for final inclusion was further determined by examining the full text.

Primary outcomes were defined as: Intraoperative bleeding volume (measured in mL). Postoperative bleeding volume at 2, 4, 6, and 24 h (measured in mL). Red blood cell (RBC) transfusion rate (percentage of patients requiring RBC transfusion postoperatively).

Secondary outcomes included transfusion volumes of RBCs, fresh frozen plasma (FFP), and PC. Postoperative 24-h laboratory parameters: platelet count, hemoglobin concentration, prothrombin time (PT), activated partial thromboplastin time (APTT), fibrinogen level, and D-dimer concentration.

Incidence of postoperative complications (e.g., mortality, myocardial infarction, arrhythmia, cerebrovascular accidents, wound infections, acute renal insufficiency, thrombotic events). Length of stay in the intensive care unit (ICU) and hospital.

All blood loss measurements were extracted directly from the original study for meta-analysis.

### 2.4 Study quality assessment

Two authors (Z. Y. Z. and Y. T. Y.) independently assessed the risk of bias using the tool described in the Cochrane Handbook for Systematic Reviews of Interventions (29). Additionally, a modified *Jadad* score (30) was used independently by two authors (Z. Y. Z. and Y. T. Y.) to evaluate the methodologic quality of each included trial.

### 2.5 Data abstraction

Two authors (Y. T. Y. and J. R. S) independently performed data extraction: (1) author, year of publication and journal of included studies; (2) total number of patients, number of patients in TXA and Control groups, gender, age; (3) data regarding outcomes of interest. Disagreements were resolved by discussion among all authors during data abstraction. One unit of RBCs ~120 mL and 1 unit of FFP ~100 mL (10).

### 2.6 Statistical analysis

All data were analyzed using RevMan 5.3 (Cochrane Collaboration, Oxford, United Kingdom). Pooled odds ratio (OR) and 95% confidence interval (CI) were estimated for dichotomous data, and weighted mean difference (WMD) and 95% CI for continuous data, respectively. Each outcome was tested for heterogeneity, and randomized-effects or fixed-effects model was used in the presence or absence of heterogeneity (Q-statistical test *p* < 0.05). Sensitivity analyses were done by examining the influence of the statistical model on estimated treatment effects, and analyses that adopt the fixed-effects model were repeated again using the randomized-effects model and vice versa. In addition, sensitivity analysis was performed to evaluate the influence of individual studies on the overall effects. If necessary, subgroup analyses were performed to evaluate the possible effects of patient characteristics and control agents on the outcomes. Publication bias was explored through visual inspection of funnel plots of the outcomes. All *p* values were two-sided, and statistical significance was *p* < 0.05.

Odds ratio (OR) was chosen because: baseline risk variability (5–20% for key outcomes) could bias risk ratio (RR), and OR is more robust to heterogeneity in multi-center data. Sensitivity analyses confirm OR and RR differ by <5% for events with >10% incidence, supporting validity. For rare events (e.g., thrombotic complications, mortality), OR approximates RR, and our sensitivity analyses using RR yielded consistent results (Supplementary Table 3).

## 3 Results

### 3.1 Search results

**Figure 1:**
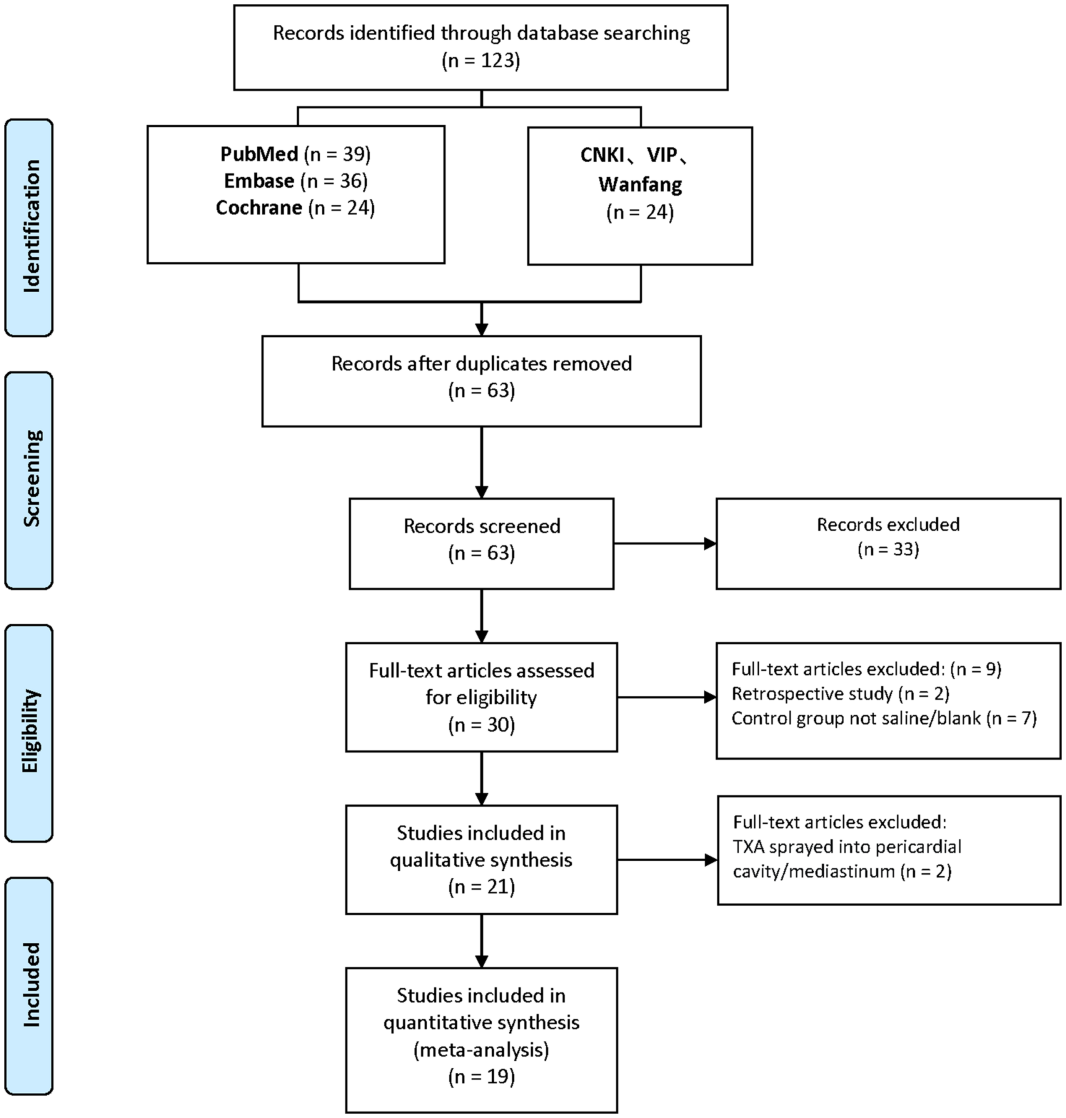
Flowchart of the included and excluded studies.

As depicted in the flow chart (Figure 1), a database search identified 30 articles for complete evaluation. Finally, 19 eligible trials (10–28) were included in the current study. Descriptive analyses of these articles are presented in Table 1. Of the 19 trials, 10 were from China (10, 15, 21–28), two from Italy (18, 20), two from Czech (17, 19), two from Iran (13, 14), and one from India (11), Korean (12), UK (16).

**Table 1:** Characteristic of tranexamic acid studies.

Studies	Country	Age (year)	Sex (F/M)	Graft (number)	APT D/C	Sample size (n) /Groups(n)	TXA regimen	Control	Transfusion triggers	Outcomes
Ahn 2012 (12)	Korea	T69 ± 9 C67 ± 7	35/41	T3.1 ± 0.6 C3.1 ± 0.6	Within 5 days	76//2	1 g ivgtt. Within 20 min before SI, 200 mg/h ivgtt. Till EOP.	Saline	RBC: Hb<85 g/dL, FFP: INR > 1.5 with bleeding PC: PLT < 75,000/mm^3^ + bleeding >200 mL/h for 2 h	3/5/6/7/8/9/10/13/14/15/20/21/22/29/30
Casati 2001 (20)	Italy	T64 ± 13 C62 ± 11	8/32	NR	D/C	40/2	1 g ivgtt. Before SI, 400 mg/h ivgtt. Till EOP.	Saline	RBC: Hb<80 g/dL, FFP: PT > 1.5 × baseline+diffuse bleeding, PC: PLT < 50,000/mm3	3/5/6/7/8/9/10/11/13/14/15/20/21/22/26/28/29
Casati 2004 (18)	Italy	T64 ± 12 C62 ± 11	12/86	NR	D/C	102/2	1 g ivgtt. Before SI, 400 mg/h ivgtt. Till EOP.	Saline	RBC: Hb<80 g/dL, FFP: PT > 1.5 × baseline, PC: PLT < 50,000/mm3	3/5/6/7/8/9/10/11/12//18/20/23/24/27
Chakravarthy 2012 (11)	India	T58 ± 4 C60 ± 6	22/78	T4.0 C3.0	≥7 days	100/2	10 mg/kg ivgtt. Over 20 min before SI, 1 mg/kg/h over the next 12 h.	Blank	RBC: Hb<90 g/dL, FFP: NR, PC: NR	1/5/9/15
Guo 2015 (24)	China	NR	17/43	T3.6 ± 0.5 C4.9 ± 0.6	NR	60/2	20 mg/kg ivgtt. When SI.	Saline	RBC: Hb<80 g/dL, FFP: NR, PC: NR	1/2/5/6/20/21/22/23/24/29
Guo 2007 (27)	China	T61 ± 8 C63 ± 8	16/60	T2.8 ± 0.5 C2.9 ± 0.4	5–7 days	76/2	750 mg ivgtt. Within 10 min after AI, 250 mg/h ivgtt. Till total 1,500 mg.	Saline	RBC: Hb<85 g/dL, FFP: NR, PC: NR	2/3/4/5/7/8/29/30
Jares 2003 (19)	Czech	NR	12/35	T1.9 ± 0.8 C1.9 ± 0.6	D/C	47/2	1 g ivgtt. Within 10 min before SI, 400 mg/h ivgtt. Till EOP.	Blank	RBC: Hb<80 g/dL, FFP: NR, PC: NR	7/10/11/12//15
Li 2017 (23)	China	T54 ± 3 C55 ± 6	13/27	NR	>7 days	40/2	20 mg/kg ivgtt. Within 30 min after AI, 10 mg/kg/h ivgtt. Till EOP.	Blank	RBC: Hb<90 g/dL, FFP: PT/APTT>1.5 normal, PC: NR	4/5/6/11/12/13/14/15/16/17/20/21/25/29/30
Mehr 2007 (14)	Iran	T44 ± 10 C45 ± 10	2/27	T2.1 ± 0.5 C2.1 ± 0.5	≥7 days	66/2	15 mg/kg ivgtt. Before SI, heparin infusion, after protamine infusion and EOP.	Saline	RBC: Hb<90 g/dL, FFP: PT > 1.5 × normal+bleeding >200 mL/h, PC: PLT < 75,000/mm^3^ + bleeding >200 mL/h for >2 h	2/4/7/8/9/10/11/13/14/15/16/17/20/21/22/24/26/29
Murphy 2006 (16)	UK	T65 ± 7 C66 ± 9	21/79	T3.0 ± 0.3 C3.0 ± 0.3	D/C	100/2	2 g ivgtt. Over 20 min after AI and before SI.	Saline	RBC: Hb<85 g/dL, FFP: At discretion of ICU staff: bleeding or coagulopathy, PC: At discretion of ICU staff: diminished PLT count	3/5/6/7/8/9/10/11/12/13/14/15/16/17/18/19/20/29
Qi 2018 (21)	China	T76 ± 5 C77 ± 5	58/282	T3.5 ± 0.7 C3.5 ± 0.7	NR	430/2	20 mg/h iv. After AI.	Saline	RBC: Hb<90 g/dL, FFP: APTT>1.5 normal, PC: PLT<50 × 109/L	4/5/7/8/9/10/29/30
Taghaddomi 2009 (13)	Iran	T55 ± 2 C60 ± 10	28/72	T3.8 C3.8	Application without detail of continuation	100/2	1 g ivgtt. Within 10 min before SI, 400 mg/h ivgtt. Till EOP.	Saline	RBC: Hb<90 g/dL, FFP: Bleeding >150 mL/h or >100 mL/h in 2 h, PC: NR	3/5/6/7/8/11/15/20/21/22/25/26/29
Vanek 2005 (17)	Czech	T68 ± 2 C69 ± 2	14/38	T1.9 ± 0.1 C1.9 ± 0.1	D/C	60/2	1 g ivgtt. Within 10 min before SI, 200 mg/h ivgtt. Till EOP.	Saline	RBC: Hb<85 g/dL, FFP: chest drain bleeding increased to 150 mL/h or to 100 mL/h for two consecutive hours, PC: NR	1/3/5/6/7/8/10/11/15/16/17/20/22/23/24/25/28
Wang 2011 (1, 26)	China	T54 ± 8 C55 ± 8	12/48	NR	NR	60/2	1 g ivgtt. Within 30 min after AI, 400 mg/h ivgtt. Till EOP.	Saline	RBC: Hb<90 g/dL, FFP: postoperative chest tube drainage volume>150 mL/h or 100 > ml/h for 2 h, PC: NR	4/5/6/7/8/16/17/20/21/24/25/27
Wang 2011 (2, 25)	China	T60 ± 8 C61 ± 8	36/195	NR	NR	260/2	1 g ivgtt. Within 30 min after AI, 400 mg/h ivgtt. Till EOP.	Saline	RBC: Hb<90 g/dL, FFP: postoperative chest tube drainage volume>150 mL/h or 100 > ml/h for 2 h, PC: NR	4/5/6/7/8/14/16/17/19/20/21/25/29
Wang 2012 (10)	China	T61 ± 8 C60 ± 9	36/116	T3.0 ± 0.8 C3.0 ± 0.8	>5 days	231/2	1 g ivgtt. Within 30 min after AI, 400 mg/h ivgtt. Till EOP.	Saline	RBC: Hb<90 g/dL, FFP: PT > 1.5 × baseline+diffuse bleeding, PC: PLT < 50,000/mm3 + diffuse bleeding	4/5/6/7/8/9/10/12/13/14/15/16/17/18/20/21/25/26/30
Wang 2017 (22)	China	T49 ± 12 C50 ± 13	22/38	NR	NR	60/2	10 mg/kg ivgtt. Within 15 min, and 2 mg/kg/h till EOP.	Saline	RBC: NR, FFP: NR, PC: NR	4/5/20/24/29
Wei 2006 (1, 28)	China	T60.4 ± 8.0 C63.2 ± 7.6	9/28	T2.5 ± 0.7 C3.0 ± 0.4	>7 days	37/2	750 mg ivgtt. Within 10 min after AI, 250 mg/h ivgtt. Till total 1,500 mg.	Saline	RBC: Hb<85 g/dL, FFP: Suspected deficiency of coagulation factors, “low” circulating volume, PC: NR	1/2/4/5//12/1314/15
Wei 2006 (2, 15)	China	T63 ± 8 C61 ± 8	16/60	T2.8 ± 0.6 C2.8 ± 0.6	5–7 days	76/2	750 mg ivgtt. Within 10 min after AI, 250 mg/h ivgtt. Till total 1,500 mg.	Saline	RBC: Hb<85 g/dL, FFP: chest drain bleeding increased to 150 mL/h or to 100 mL/h for two consecutive hours., PC: NR	2/4/5/6/7/8/11/12/13/14/15/16/17/20/24/29/30

AI, anesthesia induction; CPB, cardiopulmonary bypass; DB, double-blinded; EOP, end of operation; g, gram; iv., intravenously; ivgtt., intravenously guttae; kg, kilogram; OR, operation; RCT, randomized controlled trial; SI, skin incision; TXA, tranexamic acid; APT D/C, antiplatelet therapy discontinuation or continuation; RBC, red blood cell; FFP, fresh frozen plasma; Plt, platelets; PC, platelet concentrate; NR, not reported. Reported outcomes: 1, Intraoperative blood loss (mL); 2, Postoperative blood loss; 2 h (mL); 3, Postoperative blood loss; 4 h (mL); 4, Postoperative blood loss; 6 h (mL); 5, Postoperative blood loss 24 h (mL); 6, Postoperative 24 h hemoglobin; 7, red blood cells transfusion (%); 8, fresh frozen plasma transfusion (%); 9, platelet concentrates transfusion (%); 10, re-operation (%); 11, postoperative thrombotic complications (%); 12, postoperative mortality (%); 13, postoperative acute renal insufficiency (%); 14, postoperative cerebrovascular accident (%); 15, postoperative myocardial infarction (%); 16, Length of hospital stay (days); 17, Length of ICU stay (hours); 18, postoperative arrhythmia (%); 19, postoperative infection (%); 20, Postoperative 24 h platelets count; 21, Postoperative 24 h PT (seconds); 22, Postoperative 24 h APTT (seconds); 23, Postoperative 24 h Fibrinogen (mg/dL); 24, Postoperative 24 h D-dimer (mg/dL); 25, Postoperative 24 h INR(U); 26, Postoperative 24 h Creatinine (mg/L); 27, Postoperative 24 h IL-6 (pg/mL); 28, Postoperative 24 h CK-MB (μ/L); 29, red blood cells transfusion volume (unit); 30, fresh frozen plasma transfusion (mL).

### 3.2 Included trials characteristics

As shown in Table 1, 19 studies included patients who had undergone OPCAB surgery. Pre- and intra-operative data of these patients were presented in Table 1.

### 3.3 Risk of bias in included studies

Details regarding the performance of the studies against each domain were presented in the Risk of bias graph (Supplementary Figure 1). Additionally, a visual summary of judgments about each methodological quality item for each included trial was shown in Supplementary Figure 2. Of the 19 included trials, 15 trials had modified *Jadad* score ≥ 3 and were considered as high-quality RCTs, as show in Supplementary Table 2.

### 3.4 Intraoperative and postoperative bleeding

Details blood loss measurement methods across studies: 15 studies used chest tube drainage (10, 12, 14–16, 18–27), 3 used a combination of chest tube and gauze weighing (11, 13, 17), while 1 study did not specify the bleeding recording method (28).

**Figure 2:**
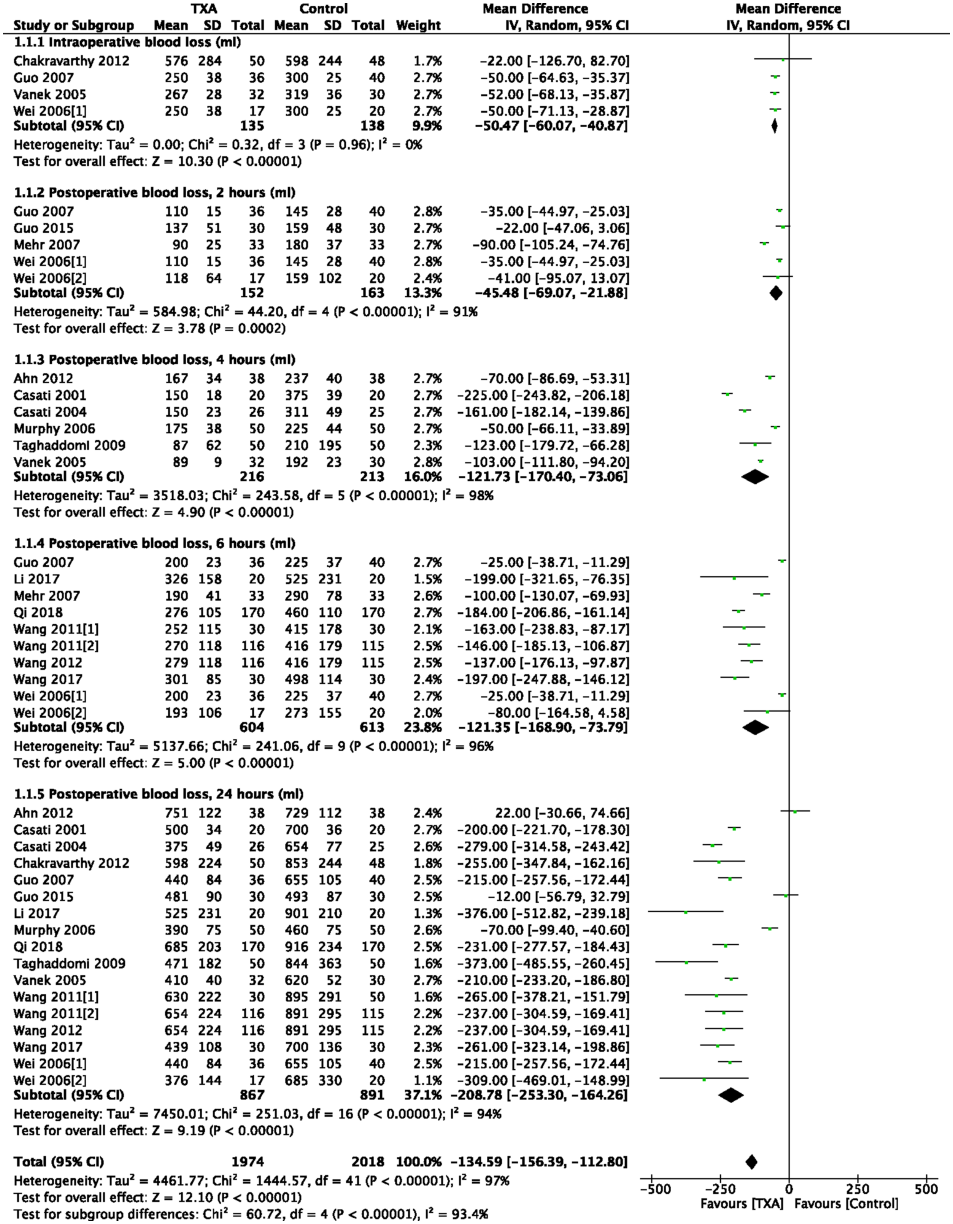
Intraoperative and postoperative bleeding.

As indicated in Table 1, 4 trials (4 comparisons, 273 patients) reported TXA reduced intraoperative bleeding volume (mL) compared to the Control group (WMD = −50.47; 95%CI: −60.07 to −40.87; *p* < 0.00001) with heterogeneity (I^2^ = 0%, *p* < 0.00001), as show in Figure 2.

As depicted in Table 1, 5 trials (5 comparisons, 315 patients), 6 trials (6 comparisons, 429 patients), 10 trials (10 comparisons, 1,217 patients), and 17 trials (17 comparisons, 1758 patients) reported the postoperative bleeding volume (ml) at 2, 4, 6, and 24 h, respectively. TXA reduced bleeding volume at 2 h post-operative compared to the Control group (WMD = −45.48; 95%CI: −69.07 to −21.88; *p* = 0.0002) with heterogeneity (I^2^ = 91%, *p* < 0.00001); TXA also reduced bleeding volume at 4 h post-operative compared to Control group (WMD = −121.73; 95%CI: −170.40 to −73.06; *p* < 0.00001) with heterogeneity (I^2^ = 98%, *p* < 0.00001); at 6 h post-operative compared to Control group (WMD = −121.35; 95%CI: −168.90 to −73.79; *p* < 0.00001) with heterogeneity (I^2^ = 96%, *p* < 0.00001); and at 24 h post-operative compared to Control group (WMD = −208.78; 95%CI: −253.30 to −164.26; *p* < 0.00001) with heterogeneity (I^2^ = 94%, *p* < 0.00001), as show in Figure 2.

### 3.5 RBC, FFP, and PC transfusion rate

**Figure 3:**
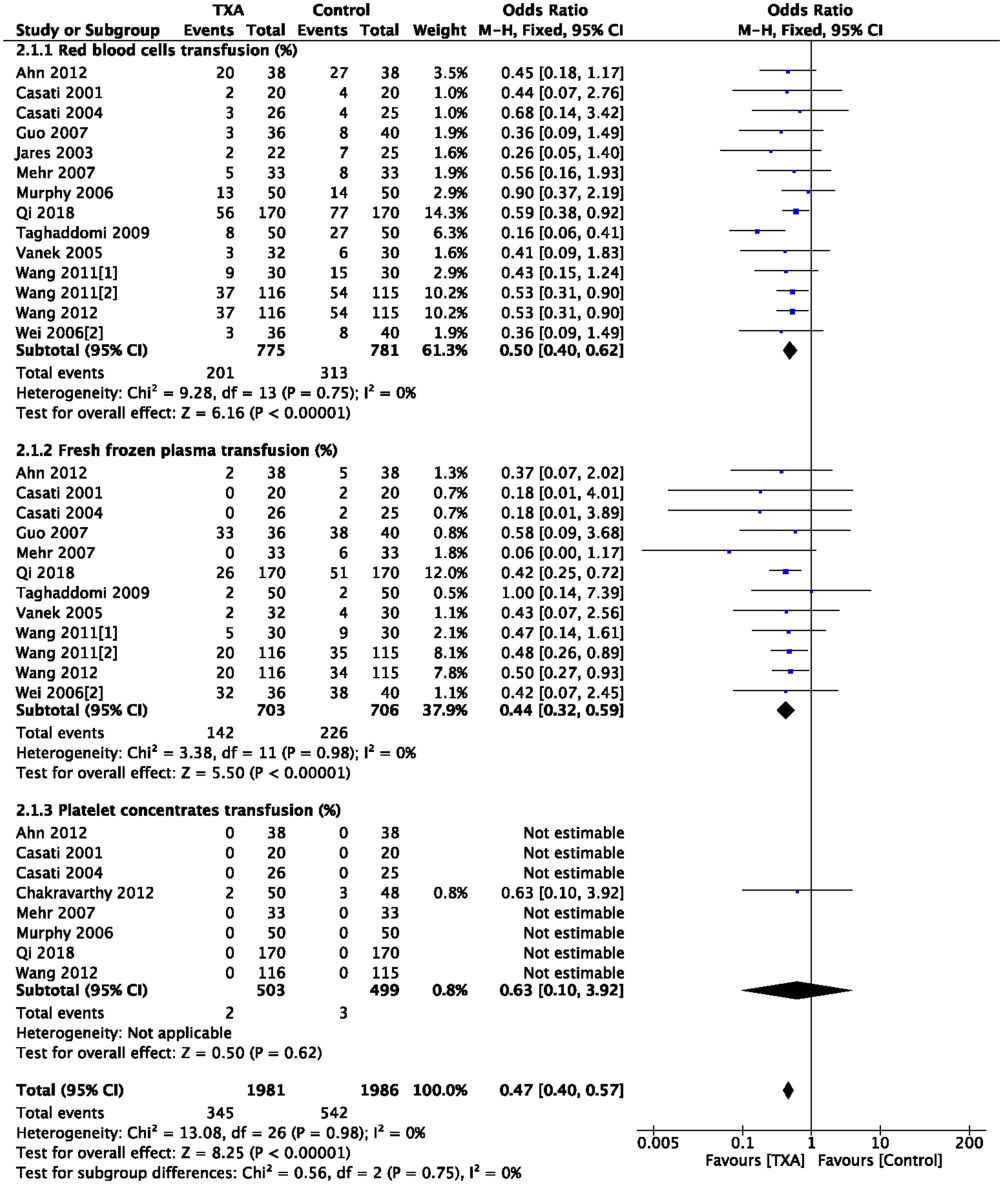
Red blood cells, fresh frozen plasma, and platelet concentrates transfusion rate.

As depicted in Table 1, 14 trials (14 comparisons, 1,556 patients), 12 trials (12 comparisons, 1,409 patients), and 8 trials (8 comparisons, 1,002 patients) reported the postoperative RBC transfusion rate, FFP transfusion rate, and PC transfusion rate, respectively. TXA reduced postoperative RBC transfusion rate compared to the Control group (OR = 0.50; 95%CI: 0.40–0.62; *p* < 0.00001) with heterogeneity (I^2^ = 0%, *p* = 0.75); TXA also reduced postoperative FFP transfusion rate compared to the Control group (OR = 0.44; 95%CI: 0.32–0.59; *p* < 0.00001) with heterogeneity (I^2^ = 0%, *p* = 0.98); PC transfusion rate was comparable between Group TXA and Group Control, as show in Figure 3.

### 3.6 RBC and FFP transfusion volume

As depicted in Table 1, 12 trials (12 comparisons, 1,265 patients) showed that TXA reduced postoperative RBC transfusion volume (unit) compared to the Control group (WMD = −1.90; 95% CI: −3.67 to −0.12; *p* = 0.0001) with high heterogeneity (I^2^ = 99%, *p* < 0.00001) (Supplementary Figure 3A). Additionally, 6 trials (6 comparisons, 839 patients) indicated that TXA reduced postoperative FFP transfusion volume (mL) compared to the Control group (WMD = −85.26; 95% CI: −150.36 to −20.16; *p* = 0.01) with heterogeneity (I^2^ = 93%, *p* < 0.00001), as show in Supplementary Figure 3B.

### 3.7 Re-operation

As shown in Table 1, 9 trials (9 comparisons, 1,013 patients) reported the incidence of postoperative reoperation for bleeding. The result of the meta-analysis suggested no difference in reoperation for postoperative bleeding between Group TXA and Group Control, as show in Supplementary Figure 4.

### 3.8 Platelet counts and hemoglobin concentrations at postoperative 24 h

**Figure 4:**
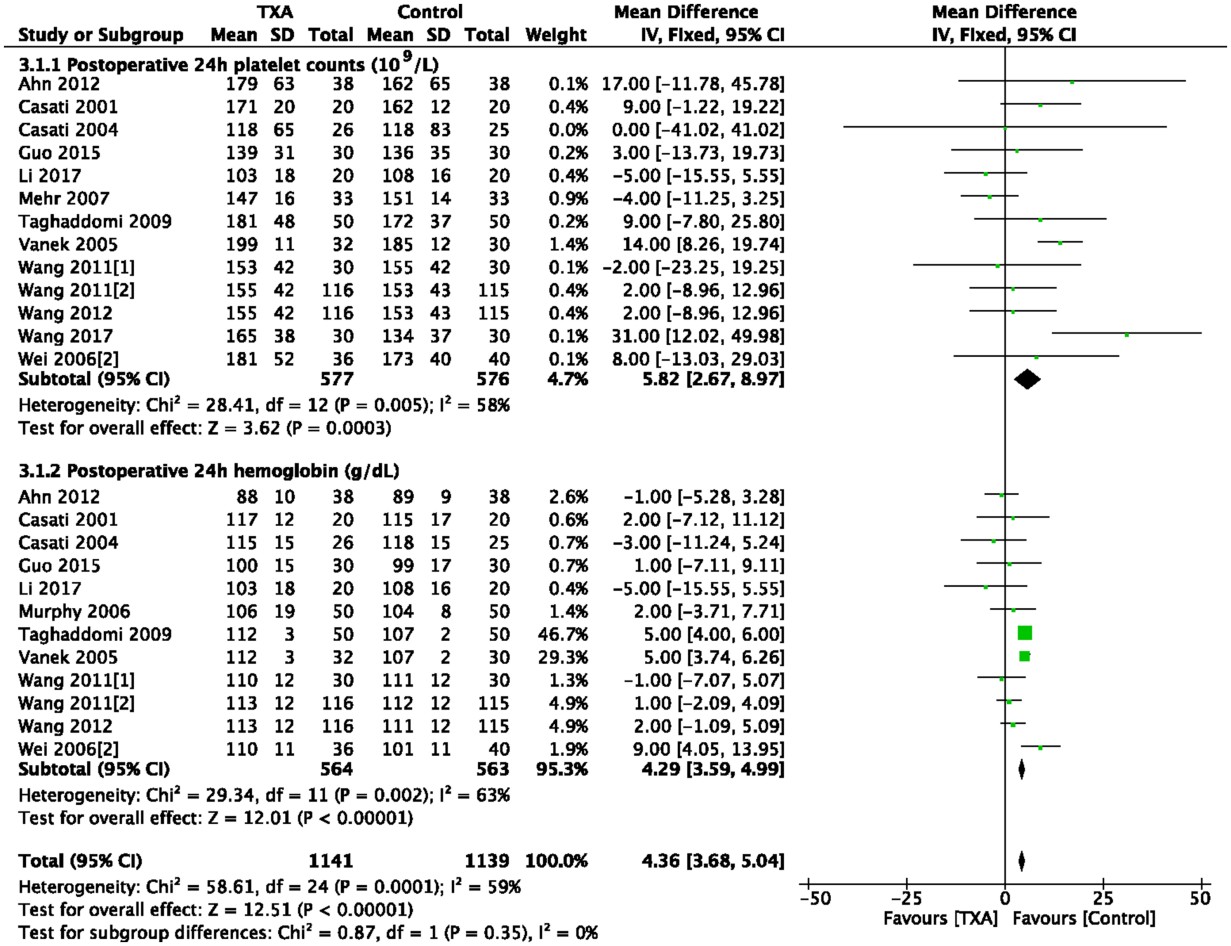
Platelet counts and hemoglobin concentrations at postoperative 24 h.

As depicted in Table 1, 13 trials (13 comparisons, 1,153 patients) reported platelet counts (10^9^/L) at postoperative 24 h. TXA increased the platelet counts compared to the Control group (WMD = 5.82; 95%CI: 2.67–8.97; *p* = 0.0003) with heterogeneity (I^2^ = 58%, *p* = 0.005; Figure 4). In total, 12 trials (12 comparisons, 1,127 patients) reported the hemoglobin concentrations (g/dL) at postoperative 24 h, TXA increased the hemoglobin concentrations compared to the Control group (WMD = 4.29; 95%CI: 3.59–4.99; *p* = 0.002) with heterogeneity (I^2^ = 63%, *p* < 0.00001), as show in Figure 4.

### 3.9 Coagulation tests at postoperative 24 h

As depicted in Table 1, 9 trials (9 comparisons, 904 patients) reported the postoperative prothrombin time (PT, seconds), TXA increased PT at postoperative 24 h compared to the Control group (WMD = 0.29; 95%CI: 0.09–0.50; *p* = 0.005) with heterogeneity (I^2^ = 32%, *p* = 0.16).

Six trials (6 comparisons, 724 patients) reported the postoperative international normalized ratio (INR, U), there is no difference in INR at postoperative 24 h between Group TXA and Group Control.

Six trials (6 comparisons, 404 patients), reported the activated partial thromboplastin time (APTT, seconds), TXA decreased APTT at postoperative 24 h compared to the Control group (WMD = −1.48; 95%CI: −2.18 to −0.79; *p* < 0.0001) with heterogeneity (I^2^ = 32%, *p* = 0.19).

Three trials (3 comparisons, 173 patients) reported the fibrinogen (mg/dL), TXA decreased fibrinogen level at postoperative 24 h compared to the Control group (WMD = −0.20; 95%CI: −0.29 to −0.11; *p* < 0.0001) with heterogeneity (I^2^ = 73%, *p* = 0.39).

**Figure 5:**
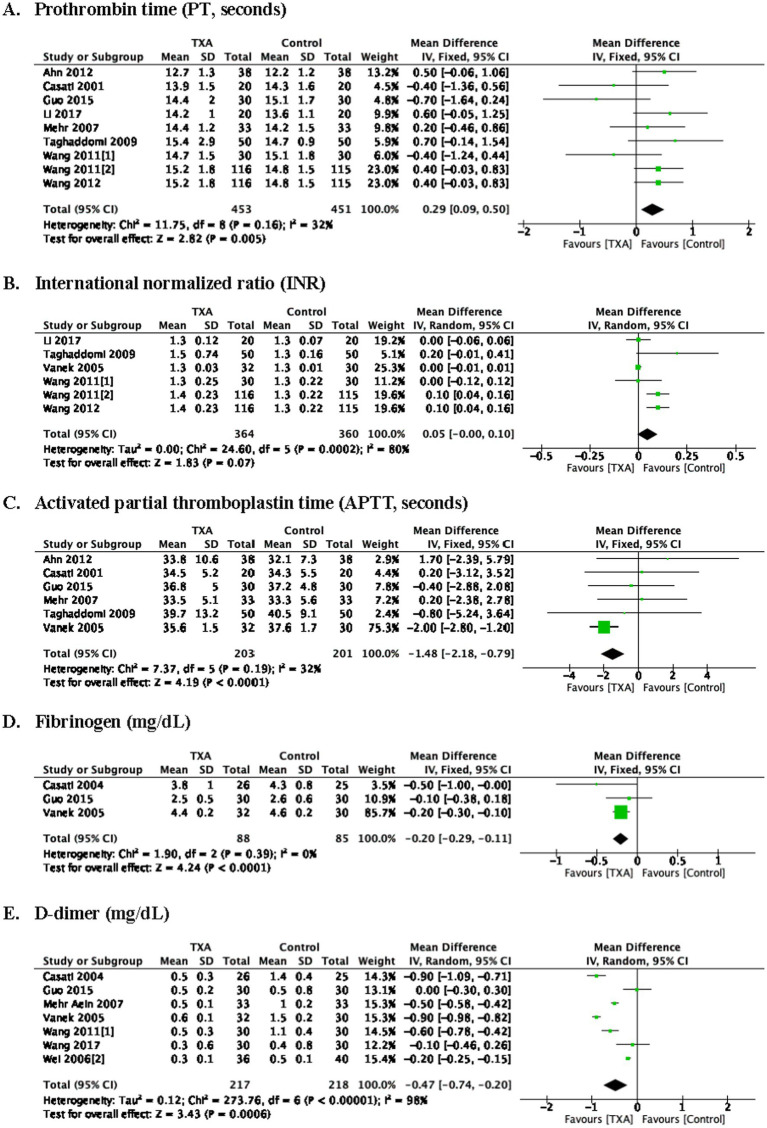
Coagulation tests at postoperative 24 h. prothrombin time **(A)**, international normalized ratio **(B)**, activated partial thromboplastin time **(C)**, fibrinogen **(D)**, D-dimer **(E)**.

Seven trials (7 comparisons, 435 patients) reported the postoperative D-dimer (mg/dL), TXA decreased D-dimer at postoperative 24 h compared to the Control group (WMD = −0.47; 95%CI: −0.74 to −0.20; *p* = 0.0006) with heterogeneity (I^2^ = 98%, *p* < 0.00001) as show in Figure 5.

### 3.10 Blood CK-MB, creatinine, and interleukin-6

As depicted in Table 1, 2 trials (2 comparisons, 94 patients), 4 trials (4 comparisons, 437 patients), and 2 trials (2 comparisons, 111 patients) reported the CK-MB (u/L), Creatinine (mg/L), and interleukin-6 (pg/mL), respectively. The CK-MB, creatinine, and interleukin-6 concentrations were comparable between Group TXA and Group Control, as show in Supplementary Figure 5.

### 3.11 Mortality

As depicted in Table 1, 7 trials (7 comparisons, 582 patients) reported the incidence of postoperative mortality (%) after OPCAB, there was no difference between Group TXA and Group Control, as show in Supplementary Figure 6.

### 3.12 Myocardial infarction and arrhythmia

Twelve trials (12 comparisons, 973 patients) reported the incidence of myocardial infarction after OPCAB surgery, 3 trials (3 comparisons, 382 patients) reported the incidence of arrhythmia after OPCAB, there was no difference between Group TXA and Group Control, as show in Supplementary Figure 6.

### 3.13 Cerebrovascular events

Eight trials (8 comparisons, 673 patients) reported the incidence of the cerebrovascular accident after OPCAB, there was no cerebrovascular accident in those trials, as show in Supplementary Figure 6.

### 3.14 Wound infection

Two trials (2 comparisons, 331 patients) reported the incidence of wound infection after OPCA BG, there was no difference between Group TXA and Group Control, as show in Supplementary Figure 6.

### 3.15 Acute renal insufficiency

Eight trials (8 comparisons, 666 patients) reported the incidence of acute renal insufficiency after OPCAB, there was no difference between Group TXA and Group Control, as show in Supplementary Figure 6.

### 3.16 Thrombotic complications

Nine trials (9 comparisons, 582 patients) reported the incidence of thrombotic complications after OPCAB, there was no difference between Group TXA and Group Control, as show in Supplementary Figure 6.

### 3.17 Length of stay in the intensive care unit and hospital

As depicted in Table 1, 8 trials (8 comparisons, 866 patients) reported the lengths of stay in the intensive care unit (hours) and lengths of stay in the hospital (days), there is no difference between Group TXA and Group Control, as show in Supplementary Figure 7.

## 4 Discussion

To our knowledge, this is the first study dedicated to more comprehensively evaluating the efficacy and safety of TXA in OPGABG surgery, focusing solely on intravenous administration. The current study demonstrated that intravenous TXA reduced RBC and FFP transfusion rates, which is clinically meaningful given the association of transfusions with increased infections and prolonged hospital stays. While TXA also decreased intraoperative and 24-h postoperative bleeding, this finding is tempered by high heterogeneity due to variable measurement methods (e.g., chest tube drainage vs. gauze weighing), limiting its clinical relevance. Notably, such blood loss differences did not correlate with improved hard outcomes like reoperation rates, reinforcing that transfusion practices—not absolute blood loss—are the primary actionable endpoint. More well-designed and adequately powered RCTs are needed to confirm this further.

TXA exerts hemostatic effects by inhibiting fibrinolysis (31). TXA altered several coagulation parameters (e.g., increased PT, decreased APTT and D-dimer) at 24 h, but these surrogate markers lack direct correlation with clinical outcomes (e.g., thrombotic events, which were similar between groups). Their utility is limited to mechanistic insights (e.g., fibrinolysis inhibition) rather than guiding patient management.

In our current analysis, we found that intravenous administration of TXA reduced the 2, 4, 6, and 24-h postoperative chest tube drainage and was associated with a lower risk of RBC and FFP transfusion in OPCAB surgery, which was consistent with the previous study (5, 6, 32). The efficacy of TXA in OPCAB was also confirmed. High heterogeneity in bleeding volume analyses reflects unreliable measurement methods, undermining the significance of 100–250 mL differences. This supports transfusion rates as the superior, more standardized endpoint. In addition, the methodological quality of this study supports the robustness of the results. Among the 19 included studies, 15 had a modified Jadad score ≥ 3 (high-quality studies, Supplementary Table 2), showing a low risk of bias in randomization and outcome assessment, as show in Supplementary Figures 1, 2.

A sensitivity analysis excluding the 4 lower-quality studies (Jadad score < 3) was added to Supplementary Table 4. The results showed no changes in key outcomes RBC transfusion rate and no differences in outcomes 24-h bleeding, confirming that lower-quality studies did not bias the overall estimates. Particularly for postoperative complications (e.g., thrombotic events), high-quality studies consistently demonstrated that TXA did not increase the risk, reducing the interference of confounding factors on the conclusions.

Two studies in this meta-analysis reported the risk of PC transfusion, but most did not report patients needing PC transfusion. Three studies with 213 participants reported reoperation for postoperative bleeding, and only one patient suffered reoperation in the TXA group and two patients in the placebo group (Figure 5). However, none of the three studies specified their criteria for reoperation. Therefore, our conclusion that reoperation for postoperative bleeding and PC transfusion rate were comparable between Group TXA and Group Control still needs to be elucidated.

Cardiac surgery (with/without CPB) involves microcirculatory alterations (33), which may impair platelet function (33, 34). TXA may reduce bleeding by preventing fibrinolytic enzyme-induced platelet activation; our previous analyses demonstrated improved ADP-stimulated platelet aggregation and CD63 expression with TXA (35).

A previous meta-analysis suggested increased seizure risk with TXA (36), potentially mediated by GABAA and glycine receptor disinhibition (37, 38). Adverse events like seizures are reported to be dose-related in previous studies (39, 40), but meta-regression or subgroup analyses could not be performed due to insufficient reporting of detailed dose data in included studies. In an RCT of 4,631 CABG patients, TXA reduced bleeding/transfusion without increasing 1-year thrombotic events or mortality (41, 42), but 100 and 50 mg/kg single doses were linked to seizures, stroke, and death (41). A multicenter RCT in cardiac surgery with CPB (9) (3,079 patients) showed that high-dose TXA modestly reduced RBC transfusion vs. low-dose, with noninferior safety (30-day mortality, seizure, kidney dysfunction, thrombotic events). Continuous TXA infusion may improve efficacy and safety vs. single doses (43), highlighting the need for optimized dosing regimens in OPCAB. Wang et al. (7) found TXA reduced CK-MB and cTnI in cardiac surgery, indicating less myocardial injury. Xie et al. (8) reported TXA reduced postoperative proinflammatory biomarkers (interleukin-6, −8, TNF-*α*) in cardiac surgery patients.

Notably, 10 of the 19 included studies were conducted in Chinese centers, prompting consideration of generalizability. As shown in Table 1, minor regional variations were observed in transfusion practices (e.g., Hb thresholds for RBC transfusion) and surgical details (e.g., graft number, antiplatelet discontinuation protocols). However, TXA’s consistent effect in reducing bleeding and transfusion rates across both Chinese and international studies supports its broader applicability. That said, generalizability may be constrained in settings with markedly different perioperative protocols—such as more liberal transfusion triggers or distinct surgical techniques. Importantly, our core finding—TXA’s efficacy in reducing transfusions without increasing adverse events—aligns with global evidence (6, 32), reinforcing its relevance beyond the included populations.”

Despite established TXA pharmacokinetics in CPB cardiac surgery (9, 41). OPCAB dosing and safety remain controversial due to limited RCTs on transfusion, bleeding, and adverse events (e.g., thrombotic events, seizures). Current results require cautious interpretation due to limited studies and high heterogeneity.

## 5 Conclusion

Intravenous TXA reduced intraoperative and postoperative bleeding volume. It also decreased the rate and volume of RBC and FFP transfusions, with no effect on reoperation rates due to postoperative bleeding. At 24 h postoperatively, TXA increased platelet counts, hemoglobin concentrations, and prothrombin time (PT), while decreasing activated partial thromboplastin time (APTT), fibrinogen levels, and D-dimer concentrations. Importantly, TXA did not elevate the risk of postoperative complications (e.g., mortality, myocardial infarction, cerebrovascular accidents, thrombotic events) and did not affect ICU and hospital stays.

### 5.1 Limitations

There are some limitations in this meta-analysis. *Heterogeneity*: High heterogeneity was observed in analyses of bleeding volume and transfusion volume, potentially attributed to variations in TXA dosing regimens, surgical techniques, transfusion thresholds, and bleeding measurement methods (e.g., chest tube drainage vs. gauze weighing). *Data Imputation*: Some included studies reported data as medians and interquartile ranges, which were converted to means and standard deviations for meta-analysis. This imputation may have introduced inaccuracies. *Dose–Response Analysis*: Due to the diversity of TXA doses (e.g., 1 g bolus, 10-20 mg/kg loading dose with maintenance infusion) and regimens across studies, subgroup analyses to evaluate dose-dependent effects (e.g., on seizures or thrombotic events) were not feasible. *Study Quality Variability*: While 15 studies were classified as high-quality (modified Jadad score ≥3), 4 lower-quality studies were included. Although sensitivity analyses confirmed their minimal impact, residual bias from methodological differences cannot be fully excluded. *Generalizability*: A substantial proportion of included studies were from Chinese centers, which may limit the generalizability of findings to populations with differing clinical practices (e.g., transfusion thresholds or surgical techniques).

## Data Availability

The original contributions presented in the study are included in the article/Supplementary material, further inquiries can be directed to the corresponding author.
