# Supplementary material for: The safety and efficacy of intravenous administration of tranexamic acid in off-pump coronary artery bypass grafting: a systematic review and meta-analysis
Source: Front Med (Lausanne). 2025 Sep 5;12:1643712. doi: 10.3389/fmed.2025.1643712 (PMC12446334; doi:10.3389/fmed.2025.1643712)
Supplement: Supplementary file 1 [file Table_1.DOCX]

**Supplement Table 1.** Search Strategy

| **PUBMED**  ("tranexamic acid"[MeSH Terms] OR ("tranexamic"[All Fields] AND "acid"[All Fields]) OR "tranexamic acid"[All Fields]) AND "off pump"[All Fields] AND " Coronary artery bypass"[All Fields]AND (Randomized Controlled Trial[ptyp] AND "humans"[MeSH Terms]) |
| --- |
| **EMBASE**  #1 'tranexamic acid'/exp OR 'tranexamic acid':ab,ti  #2''off pump coronary artery bypass'/exp  #3'randomized controlled trial'/exp OR 'controlled randomized trial'/exp  #1 AND #2 AND #3  ('tranexamic acid'/exp OR 'tranexamic acid' OR 'tranexamic acid':ab,ti) AND (off pump coronary artery bypass '/exp) AND ('randomized controlled trial'/exp OR 'randomized controlled trial' OR 'controlled randomized trial') |
| **OVID**  (tranexamic acid). ab. AND (platelet).ab. AND (off pump coronary artery bypass).tw. AND randomized.ab |
| **China National Knowledge Infrastructure (CNKI)**  (off pump coronary artery bypass [All field]) AND (Tranexamic Acid [All field]) AND (Randomized controlled trial[pytp] OR controlled clinical trial[pytp] OR randomized [All field] OR placebo [All field] OR randomly [All field] OR trial [All field]) |
| **Wanfang Data**  (off pump coronary artery bypass [All field]) AND (Tranexamic Acid [All field]) AND (Randomized controlled trial[pytp] OR controlled clinical trial[pytp] OR randomized [All field] OR placebo [All field] OR randomly [All field] OR trial [All field]) |
| **VIP Date**  (off pump coronary artery bypass [All field]) AND (Tranexamic Acid [All field]) AND (Randomized controlled trial[pytp] OR controlled clinical trial[pytp] OR randomized [All field] OR placebo [All field] OR randomly [All field] OR trial[All field]) |
