# Supplementary material for: The safety and efficacy of intravenous administration of tranexamic acid in off-pump coronary artery bypass grafting: a systematic review and meta-analysis
Source: Front Med (Lausanne). 2025 Sep 5;12:1643712. doi: 10.3389/fmed.2025.1643712 (PMC12446334; doi:10.3389/fmed.2025.1643712)
Supplement: Supplementary file 2 [file Table_2.DOCX]

**Supplement Table 2.** Quality assessment of included studies

| Study | Sample size | *Jadad* Score | | | |
| --- | --- | --- | --- | --- | --- |
|  |  | Randomization | Blindness | Withdrawals | Total |
| Ahn 2012 ^[12]^ | 76 | 2 | 2 | 1 | 5 |
| Casati 2001 ^[20]^ | 40 | 1 | 2 | 1 | 4 |
| Casati 2004 ^[18]^ | 102 | 2 | 2 | 1 | 5 |
| Chakravarthy 2012 ^[11]^ | 100 | 1 | 0 | 1 | 2 |
| Guo 2015 ^[26]^ | 60 | 1 | 0 | 1 | 2 |
| Guo 2007 ^[29]^ | 76 | 2 | 1 | 0 | 3 |
| Jares 2003 ^[19]^ | 47 | 1 | 1 | 1 | 3 |
| Li 2017 ^[24]^ | 40 | 2 | 1 | 1 | 4 |
| Mehr Aein 2007 ^[14]^ | 66 | 2 | 2 | 1 | 5 |
| Murphy 2006 ^[16]^ | 100 | 2 | 2 | 1 | 5 |
| Qi 2018 ^[22]^ | 430 | 2 | 1 | 1 | 4 |
| Taghaddomi 2009 ^[13]^ | 100 | 2 | 2 | 1 | 5 |
| Vanek 2005 ^[17]^ | 60 | 2 | 2 | 1 | 5 |
| Wang 2011[1] ^[28]^ | 60 | 2 | 2 | 1 | 5 |
| Wang 2011[2] ^[27]^ | 260 | 2 | 2 | 1 | 5 |
| Wang 2012 ^[10]^ | 231 | 2 | 2 | 1 | 5 |
| Wang 2017 ^[23]^ | 60 | 2 | 2 | 1 | 5 |
| Wei 2006[1] ^[30]^ | 37 | 1 | 0 | 1 | 2 |
| Wei 2006[2] ^[15]^ | 76 | 1 | 0 | 1 | 2 |
