# Supplementary material for: The safety and efficacy of intravenous administration of tranexamic acid in off-pump coronary artery bypass grafting: a systematic review and meta-analysis
Source: Front Med (Lausanne). 2025 Sep 5;12:1643712. doi: 10.3389/fmed.2025.1643712 (PMC12446334; doi:10.3389/fmed.2025.1643712)
Supplement: Supplementary file 3 [file Table_3.DOCX]

**Supplement Table 3.** Influence of statistical model on the outcomes.

|  | **Statistical model** | |
| --- | --- | --- |
| **Outcomes** | **Fixed Effects**  **WMD [95% CI]** | **Random Effects**  **WMD [95% CI]** |
| Intraoperative blood loss [ml] | -50.47 [-60.07, -40.87] | -50.47 [ -60.07 to -40.87] |
| Postoperative blood loss, 2 hours [ml] | -43.27[-49.43, -37.12] | -45.48 [-69.07, -21.88] |
| Postoperative blood loss, 4 hours [ml] | -109.13[-115.37, -102.89] | -121.73 [-170.40, -73.06] |
| Postoperative blood loss, 6 hours [ml] | -66.25[-74.22, -58.28] | -121.35 [-168.90, -73.79] |
| Postoperative blood loss, 24 hours [ml] | -184.96 [-195.27, -174.66] | -208.78 [-253.30 to -164.26] |
| RBC transfusion rate [%] | 0.50 [0.40, 0.62] | 0.50 [0.40, 0.63] |
| FFP transfusion rate [%] | 0.44 [0.32, 0.59] | 0.45 [0.33, 0.60] |
| PC transfusion rate [%] | 0.63 [0.10, 3.92] | 0.63 [0.10, 3.92] |
| RBC transfusion volume [uint] | -0.20 [-0.35, -0.06] | -1.90 [-3.67, -0.12] |
| FFP transfusion volume [ml] | -28.22 [-41.43, -15.02] | -85.26 [-150.36, -20.16] |
| Effects on re-operation [%] | 0.74 [0.14, 3.83] | 0.71 [0.11, 4.63] |
| Effects on platelet counts postoperative 24 hours [10^9^/L] | 5.82 [2.67, 8.97] | 5.59 [0.00, 11.17] |
| Effects on hemoglobin concentrations postoperative 24 hours [g/dL] | 4.29 [3.59, 4.99] | 2.71 [1.05, 4.38] |
| Postoperative prothrombin time [PT, seconds] | 0.29 [0.09, 0.50] | 0.26 [-0.00, 0.52] |
| International normalized ratio [INR, U] | 0.01 [-0.00, 0.02] | 0.05 [-0.00, 0.10] |
| Activated partial thromboplastin time [APTT, seconds] | -1.48 [-2.18, -0.79] | -0.81 [-2.01, 0.40] |
| Fibrinogen [mg/dL] | -0.20 [-0.29, -0.11] | -0.20 [-0.29, -0.11] |
| D-dimer[mg/dL] | -0.41 [-0.44, -0.38] | -0.47 [-0.74, -0.20] |
| CK-MB [u/L] | -0.15 [-0.30, -0.00] | -0.25 [-0.63, 0.13] |
| Creatinine [mg/L] | 0.00 [-0.03, 0.03] | 0.00 [-0.03, 0.03] |
| Interleukin-6 [pg/mL] | -13.20 [-20.19, -6.20] | -11.60 [-40.02, 16.81] |
| Postoperative mortality [%] | 0.96 [0.06, 16.23] | 0.96 [0.06, 16.23] |
| Myocardial infarction [%] | 1.04 [0.21, 5.28] | 1.05 [0.18, 6.24] |
| Arrhythmia [%] | 0.42 [0.13, 1.41] | 0.43 [0.13, 1.46] |
| Incidence of cerebrovascular accident | Not estimable | Not estimable |
| Wound infection [%] | \| 2.04 [0.18, 23.27] \| \| --- \| | 2.04 [0.18, 23.27] |
| Acute renal insufficiency [%] | \| 1.00 [0.33, 3.02] \| \| --- \| | \| 1.00 [0.32, 3.16] \| \| --- \| |
| Thrombotic complications [%] | \| 0.56 [0.07, 4.38] \| \| --- \| | \| 0.58 [0.07, 4.90] \| \| --- \| |
| Lengths in the intensive care unit [hours] | -0.15 [-0.39, 0.09] | -1.17 [-3.01, 0.67] |
| Lengths in the hospital [hours] | -0.04 [-0.13, 0.06] | -0.07 [-0.23, 0.09] |

Abbreviations: WMD= Weighted mean difference, OR= odds ratio, 95%CI= 95%confidence interval.
