# Supplementary material for: The safety and efficacy of intravenous administration of tranexamic acid in off-pump coronary artery bypass grafting: a systematic review and meta-analysis
Source: Front Med (Lausanne). 2025 Sep 5;12:1643712. doi: 10.3389/fmed.2025.1643712 (PMC12446334; doi:10.3389/fmed.2025.1643712)
Supplement: Supplementary file 4 [file Table_4.DOCX]

**Supplement Table 4.** Sensitivity analyses of high heterogeneity outcomes.

| **Outcomes** | **Excluded trials** | **Group T**  **(n)** | **Group C**  **(n)** | **Heterogeneity** | | **Analysis model** | **WMD/OR** | **(95%CI)** | **Overall effect *P*** |
| --- | --- | --- | --- | --- | --- | --- | --- | --- | --- |
|  |  |  |  | ***I^2^* (%)** | ***P*** |  |  |  |  |
| Intraoperative blood loss (ml) | Chakravarthy 2012 | 50 | 48 | 0 | P＜0.00001 | IV, Fixed | -50.71 | [-60.36, -41.07] | P＜0.00001 |
| Postoperative blood loss, 2 hours (ml) | Mehr 2007 | 33 | 33 | 0 | P = 0.80 | IV, Random | -34.16 | [-40.89, -27.42] | P＜0.00001 |
| Postoperative blood loss, 4 hours (ml) | Casati 2001 | 20 | 20 | 95 | P＜0.00001 | IV, Random | -99.42 | [-134.19, -64.65] | P＜0.00001 |
| Postoperative blood loss, 6 hours (ml) | Qi 2018 | 170 | 170 | 94 | P＜0.00001 | IV, Random | -111.12 | [-152.17, -70.07] | P＜0.00001 |
| Postoperative blood loss, 24 hours (ml) | Qi 2018 | 170 | 170 | 94 | P＜0.00001 | IV, Random | -184.96 | [-195.27, -174.60] | P＜0.00001 |
| RBC transfusion rate (%) | Qi 2018 | 170 | 170 | 9 | P=0.35 | IV, Fixed | 0.52 | [0.42, 0.65] | P＜0.00001 |
|  | Taghaddomi 2009 | 50 | 50 | 0 | P=0.82 | IV, Fixed | 0.41 | [0.31, 0.54] | P＜0.00001 |
| FFP transfusion rate (%) | Mehr Aein 2007 | 33 | 33 | 0 | P=1.0 | IV, Fixed | 0.43 | [0.32, 0.56] | P＜0.00001 |
| RBC transfusion volume (U) | Murphy 2006 | 50 | 50 | 99 | P＜0.00001 | IV, Random | -0.92 | [-1.33, -0.51] | P＜0.00001 |
|  | Taghaddomi 2009 | 50 | 50 | 96 | P＜0.00001 | IV, Random | 0.02 | [-0.20,0.25] | P=0.83 |
| FFP transfusion volume (ml) | Murphy 2006 | 50 | 50 | 93 | P=0.01 | IV, Random | -80.44 | [-145.01, -15.87] | P=0.01 |
| Effects on platelet counts postoperative 24 hours (10^9^/L) | Wang 2017 | 30 | 30 | 44 | P=0.04 | IV, Random | 5.51 | [0.13,10.89] | P=0.10 |
| Postoperative prothrombin time (PT, seconds) | Guo 2015 | 30 | 30 | 5 | P=0.39 | IV, Fixed | 0.30 | [0.11, 0.50] | P=0.003 |
|  | Mehr Aein 2007 | 33 | 33 | 37 | P=0.13 | IV, Fixed | 0.27 | [0.07, 0.47] | P=0.009 |
| International normalized ratio (INR, U) | Vanek 2005 | 32 | 30 | 76 | P=0.0004 | IV, Random | 0.04 | [-0.00,0.08] | P=0.07 |
| Activated partial thromboplastin time (APTT, seconds) | Vanek 2005 | 32 | 30 | 0 | P=0.55 | IV, Fixed | 0.68 | [-0.55,1.91] | P=0.28 |
| D-dimer(mg/dL) | Wei 2006[2] | 36 | 30 | 98 | P＜0.00001 | IV, Random | -0.47 | [-0.74, -0.20] | P＜0.00001 |
| Lengths in the intensive care unit (hours) | Wei 2006[2] | 36 | 30 | 0 | P=0.96 | IV, Fixed | -1.59 | [3.41,0.24] | P=0.92 |

Abbreviations: T= Tranexamic acid, C= Control, WMD=Weighted mean difference, OR= odds ratio, 95%CI= 95%confidence interval, post-op =post-operative, pre-op =pre-operative RBC=red blood cell, FFP=fresh frozen plasma, u=unit, ml=milliliter, dL=deciliter, g=gram, mg=milligram.
