# Supplementary figures and images for: The safety and efficacy of intravenous administration of tranexamic acid in off-pump coronary artery bypass grafting: a systematic review and meta-analysis
Source: Front Med (Lausanne). 2025 Sep 5;12:1643712. doi: 10.3389/fmed.2025.1643712 (PMC12446334; doi:10.3389/fmed.2025.1643712)

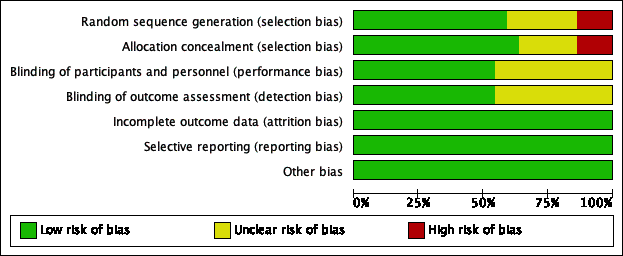

Supplement: Supplementary file 6 [file Image_1.PNG]

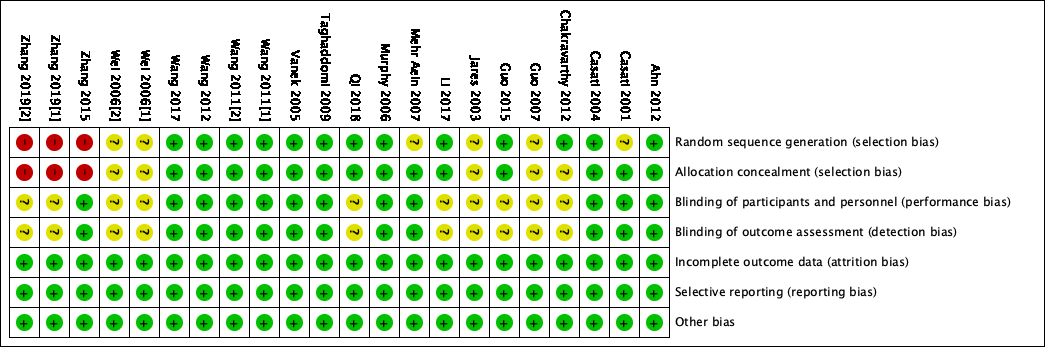

Supplement: Supplementary file 7 [file Image_2.PNG]

## A. Red blood cells transfusion (U)

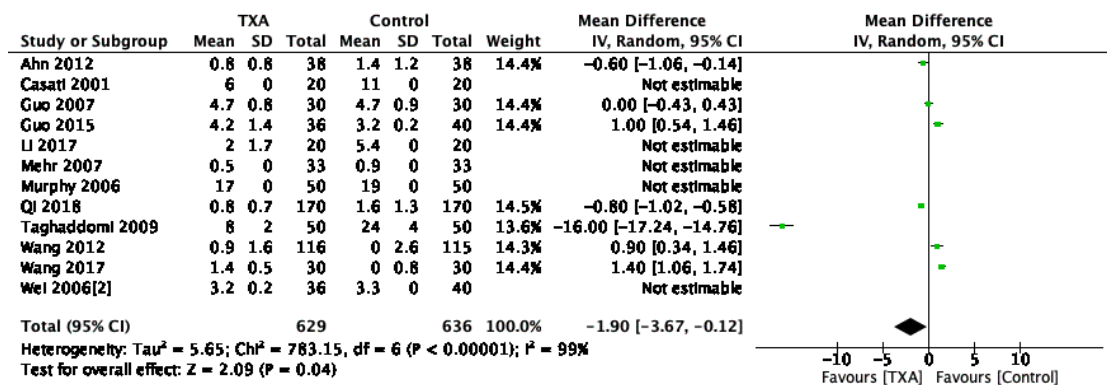

## B. Fresh frozen plasma transfusion (ml)

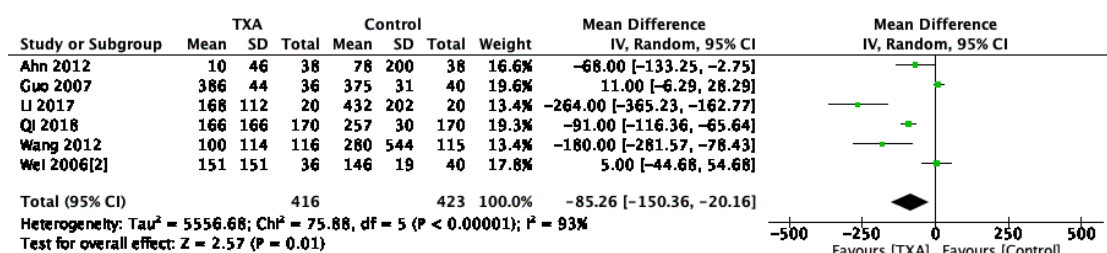

Supplement: Supplementary file 8 [file Image_3.PDF]

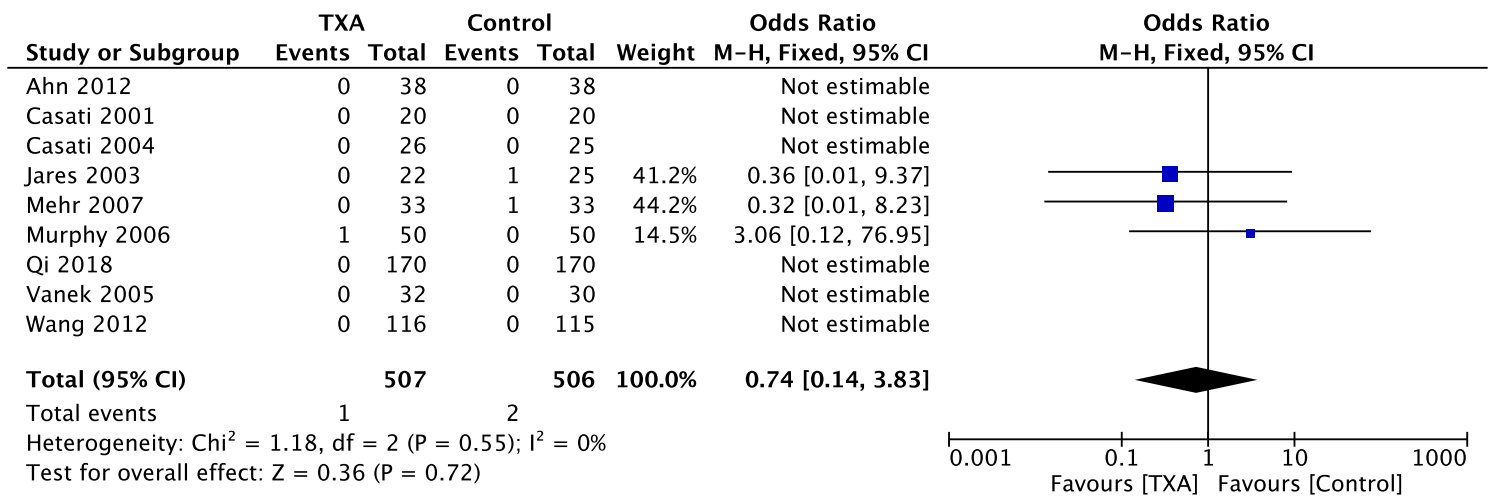

Supplement: Supplementary file 9 [file Image_4.PDF]

### A. Postoperative CK-MB (u/L)

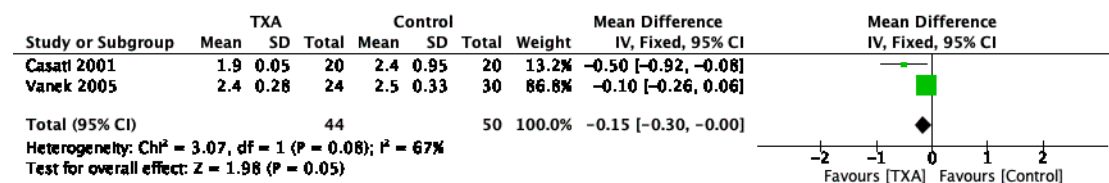

### B. Postoperative 24h Creatinine (mg/L)

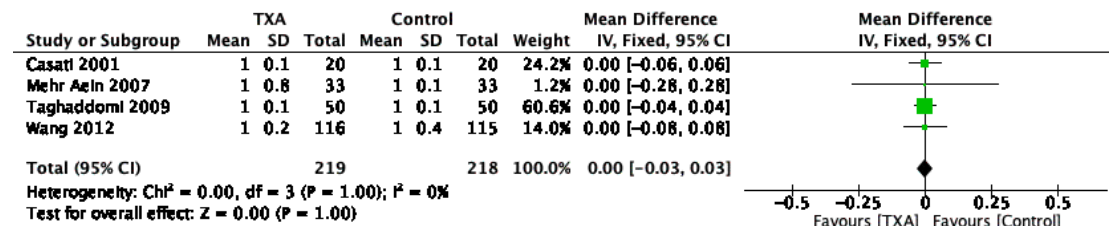

### C. Postoperative 24h interleukin-6 (pg/mL)

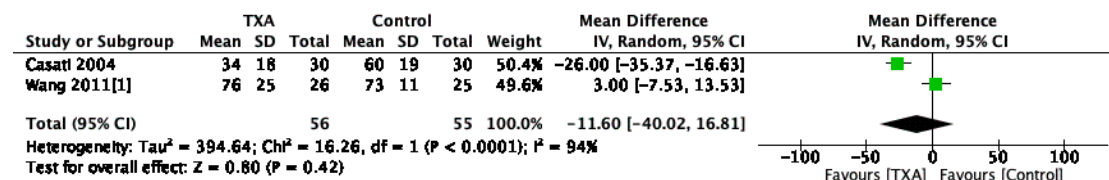

Supplement: Supplementary file 10 [file Image_5.PDF]

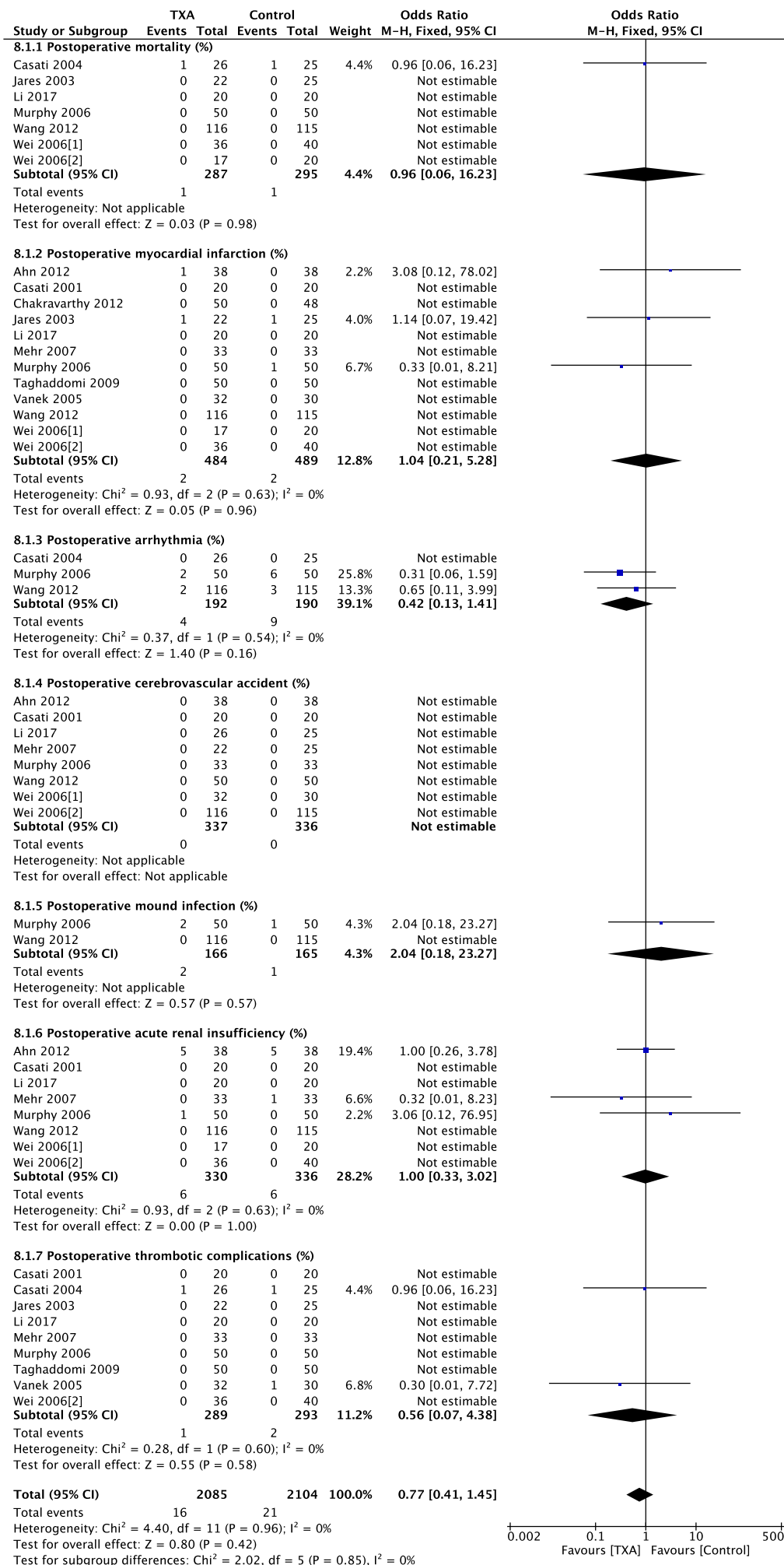

Supplement: Supplementary file 11 [file Image_6.PDF]

## A. Length of ICU stay (hours)

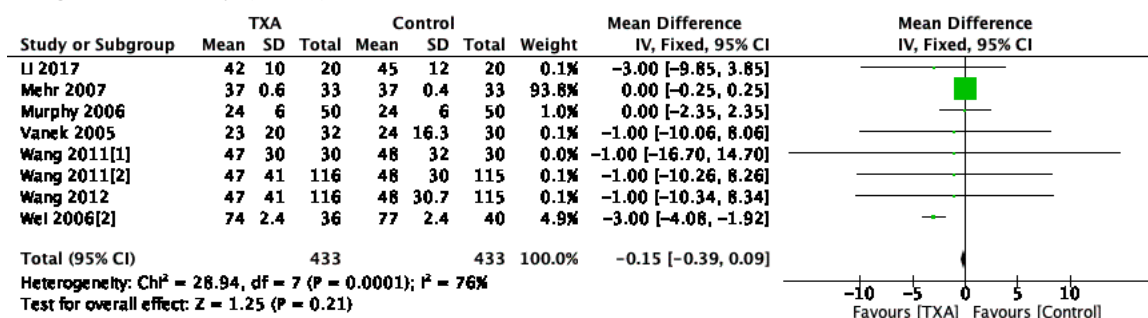

## B. Length of hospital stay (days)

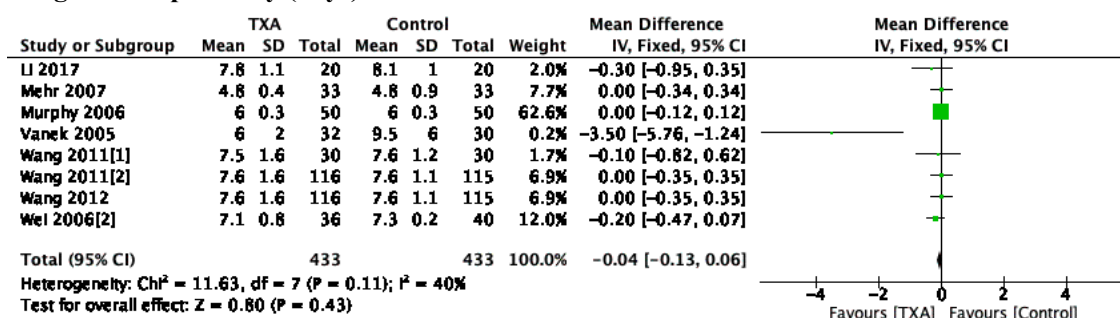

Supplement: Supplementary file 12 [file Image_7.PDF]
